# Activated Clotting Time and Haemostatic Complications in Patients Receiving ECMO Support: A Systematic Review

**DOI:** 10.3390/jcdd12070267

**Published:** 2025-07-13

**Authors:** Daniel Schwaiger, Lukas Schausberger, Benedikt Treml, Dragana Jadzic, Nicole Innerhofer, Christoph Oberleitner, Zoran Bukumirić, Igor Spurnić, Sasa Rajsic

**Affiliations:** 1 Department of Anaesthesiology and Intensive Care, Medical University of Innsbruck, Anichstraße. 35, 6020 Innsbruck, Austria; daniel.schwaiger@i-med.ac.at (D.S.);; 2 Anaesthesia and Intensive Care Department, Pain Therapy Service, Cagliari University, 09100 Cagliari, Italy; 3 Institute of Medical Statistics and Informatics, Faculty of Medicine, University of Belgrade, 11000 Belgrade, Serbia; 4 Surgical Oncology Clinic, Institute of Oncology and Radiology of Serbia, 11000 Belgrade, Serbia

**Keywords:** activated clotting time, monitoring, ACT, anticoagulation, extracorporeal membrane oxygenation, ECMO, adverse events, thrombosis, bleeding

## Abstract

**Background**: Extracorporeal membrane oxygenation (ECMO) requires systemic anticoagulation to prevent clotting, typically using unfractionated heparin (UFH). However, anticoagulation carries a bleeding risk, necessitating monitoring. Activated clotting time (ACT) is a commonly used monitoring tool for UFH anticoagulation. However, systematized evidence linking ACT monitoring with haemostatic complications (bleeding and thrombosis) is missing. **Methods**: A systematic review (Scopus and PubMed, up to 13 July 2024) including studies reporting on the patients receiving ECMO support with UFH anticoagulation monitored using ACT was performed. **Results**: A total of 3536 publications were identified, of which 30 (2379 patients) were included in the final review. Thirteen studies found no significant association between ACT values and haemorrhage, while four studies suggested a relationship between elevated ACT levels and bleeding events. Eight studies demonstrated no association between ACT values and the occurrence of thrombosis. Major bleeding was most common (49%, 13 studies with 501 events), while the pooled rate of thrombosis was 25% (16 studies with 309 events) and in-hospital mortality was 51% (17 studies, 693/1390 patients). **Conclusions**: Despite advancements in ECMO, the optimal approach for anticoagulation monitoring remains undefined. Most studies in this review did not establish a significant relationship between ACT levels and haemostatic complications. Based on the current evidence, ACT does not appear to be a reliable tool for monitoring anticoagulation in patients receiving ECMO, and alternative methods should be considered.

## 1. Introduction

The use of extracorporeal membrane oxygenation (ECMO) in patients with circulatory or respiratory failure has increased significantly in recent years. The contact of blood with artificial surfaces of the ECMO system may lead to increased clot formation with the need for systemic anticoagulation. However, the use of anticoagulation increases the risk of bleeding [1,2,3]. To mitigate the risk of haemostatic complications, appropriate anticoagulation monitoring is essential [2,4,5].

Unfractionated heparin (UFH) remains the standard for therapeutic anticoagulation in patients receiving ECMO support [6]. The Extracorporeal Life Support Organization (ELSO) recommends using various time-dependent monitoring tests (activated partial thromboplastin time—aPTT; activated clotting time—ACT), specific assays (i.e., anti-factor Xa assay), or viscoelastic testing (i.e., rotational thromboelastometry—ROTEM; thromboelastography—TEG) [6]. The International Society on Thrombosis and Haemostasis (ISTH) prioritize the use of anti-factor Xa for UFH monitoring, with ACT and aPTT as alternatives [7].

The ACT is a whole-blood test that measures the time required for clot formation. It remains the primary method for monitoring heparinization where high concentrations of UFH are present, such as during cardiac surgery, in the cardiac catheterization laboratory, during ECMO, in vascular surgery, and during dialysis, where aPTT and prothrombin time (PT) cannot be adequately measured. However, factors such as platelet count and function, hypothermia, haemodilution, and technical issues can influence the reliability of ACT, potentially limiting its utility in certain cases [4,6]. While aPTT and anti-factor Xa assays are gaining popularity as monitoring tools, ACT remains a widely utilized and significant method globally due to its rapid availability, point-of-care applicability, and low cost [8,9].

The role of ACT in monitoring of anticoagulation in patients receiving ECMO remains unclear. Moreover, systematic evidence regarding the relationship between anticoagulation guided using ACT and haemorrhagic or thromboembolic complications is lacking. Therefore, the aim of this work is to systematically evaluate the association between ACT monitoring and haemostatic complications during ECMO support.

## 2. Materials and Methods

A systematic review of the literature on anticoagulation monitoring using ACT during ECMO support was performed. This review was conducted in accordance with PRISMA guidelines (Supplementary Table S1), and the study protocol is registered in the PROSPERO database (CRD42023448888) [10].

The main aim of this review was to systemize the available evidence on the relationship between ACT monitoring and haemostatic complications (bleeding and thrombosis) in patients receiving ECMO support. Additionally, we aimed to investigate the incidence of complications and mortality rates reported in the included studies. The study inclusion and exclusion criteria are provided in Supplementary Table S2.

### 2.1. Search Strategy

A systematic search of the literature was performed in the PubMed and Scopus databases (up to 13 July 2024). The search comprised terms related to anticoagulation monitoring, ECMO support, and complications (Supplementary Table S3). All articles reporting on (a) ECMO support, (b) ACT-based monitoring, and (c) haemostatic complications were included. We excluded review articles, duplicate publications, studies lacking information on anticoagulation monitoring, and articles reporting results on the same patient population. The study limitations are provided in Supplementary Table S2.

The screening was conducted by three independent researchers (S.R., C.O., and D.J.).

### 2.2. Data Synthesis and Extraction

Two authors (D.S. and L.S.) independently performed the data extraction with a summary presented in Supplementary Table S4. Basic study information (author, country, centre, year of publication main aim, etc.), patient demographics (age, population, sex, type of ECMO-support, duration of ECMO support, anticoagulation monitoring, etc.), complications (total number of bleeding events, including various types of bleeding; thrombotic complications, including ischemic stroke, cannula-associated thrombosis, ECMO circuit, and membrane clot; renal replacement therapy, acute kidney injury, and sepsis); and mortality in different periods were collected. To standardize the results, simple calculations were performed to facilitate the comparison between studies (calculating the sex proportion, converting percentages into original values, and summing outcomes of interest). All calculations were performed separately by two authors (D.S. and L.S.).

For the pooled estimate of single proportions in the case of adverse events, inverse variance methods with logit transformation were used. We explored heterogeneity by τ2 statistics and Cochran’s Q test, quantifying with the I2 statistic.

### 2.3. Quality Assessment of Studies

The methodical quality of the articles was evaluated with the Newcastle–Ottawa Scale (NOS) [11]. Articles that scored seven or more stars on the NOS were classified as high quality, while those with at least five stars were categorized as fair quality. Studies scoring below five stars were considered low quality. 

## 3. Results

### 3.1. Search Results and Description of Studies

**Figure 1:**
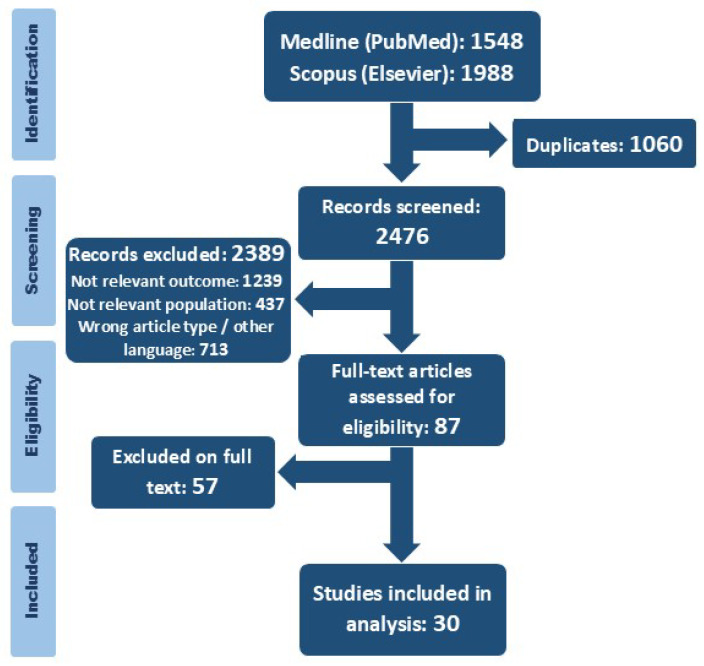
PRISMA flow chart: study identification and selection process.

The initial search resulted in 3536 publications in Medline (PubMed) and Scopus (Elsevier). After the removal of duplicates, 2476 articles were selected for titles and abstract screening (Figure 1). In the first step, 2389 articles were excluded, and 87 studies underwent full-text assessment. Finally, our systematic review comprised 30 publications.

The characteristics of the included works are presented in Table 1. The analysed works encompass data primarily from the USA (n = 23), China (n = 2), and one each from Australia, Germany, Korea, Vietnam, and Saudi Arabia. Among these studies, sixteen reported on both venoarterial (VA) and venovenous (VV) ECMO support, five exclusively on VA ECMO, and two on VV ECMO. In seven studies, the ECMO configuration was not specified.

### 3.2. Patient Population and Outcomes

This systematic review comprised data from 2379 ECMO patients in the period between 1992 and 2020. Anticoagulation was primarily utilized with UFH in all studies, with target ACT levels varying from 130 to 240 s (Supplementary Table S5).

### 3.3. Haemorrhagic Events and ACT

Seventeen studies reported on the relationship between ACT and haemorrhage, with four of them showing higher ACT levels in patients experiencing bleeding events (Supplementary Table S5). Sixteen studies reported on the superiority of other methods such as anti-factor Xa or aPTT compared to ACT, while two were neutral, as there were no differences in the use of aPTT or anti-factor Xa compared to ACT (Table S5).

### 3.4. Thromboembolic Events and ACT

Nine studies investigated the relationship between ACT and thrombosis. Eight studies did not find an association between ACT and thrombosis, while one study reported on significantly lower ACT values in patients with thrombosis [29]. Two studies reported on higher ACT values in patients with thrombosis [24,35] (Supplementary Table S5). Niebler et al. compared ACT and anti-factor Xa, reporting fewer thrombotic complications in patients guided with anti-factor Xa compared to ACT [31]. Northam et al. compared the multimodal approach with ACT and did not find any differences in thrombosis rates between protocols [32]. No other authors reported any association of ACT monitoring with thrombosis.

### 3.5. Adverse Events

The most prevalent complication observed was major bleeding, occurring in 49% of cases, followed by any form of bleeding at 48% (Table 2). Data on thrombosis were available from 16 studies, with a pooled rate of 25%. The most commonly reported thrombotic events were ECMO circuit and membrane clots, with an incidence of 17% (Table 2).

Data on in-hospital mortality were available for 1390 patients, with 693 not surviving until hospital discharge, resulting in a pooled rate of 51% (Table 2).

## 4. Discussion

This systematic review of the literature aimed to investigate the association of ACT-guided anticoagulation with haemostatic complications in patients receiving ECMO support. We included 30 studies with a total of 2379 patients, making this the most comprehensive assessment of ACT-guided anticoagulation in ECMO patients to date. The vast majority of the included studies did not find any relationship between ACT values and haemorrhage or thrombosis, implying a controversial role of ACT.

### 4.1. Monitoring of Anticoagulation During ECMO

Systemic anticoagulation remains the standard of care for preventing thrombosis and circuit clotting, but clear evidence on the optimal strategy is still lacking [2,35,42,43]. International recommendations for anticoagulation monitoring include the use of time-based tools (ACT or aPTT), viscoelastic methods, or specific coagulation assays. ACT remains a commonly used test for point-of-care anticoagulation monitoring in patients receiving ECMO [2]. Bembea et al. demonstrated that 97% of centres used ACT [44], while a follow-up analysis conducted in 2020 across 273 centres in 50 countries found that one-third of centres still use ACT. The most commonly used approaches included aPTT in more than 40% of centres and anti-factor Xa in 22% [8].

A recent meta-analysis of nine studies revealed no significant association between ACT-guided anticoagulation monitoring and the rate of haemostatic complications [45]. However, this work was limited by a small number of mostly retrospective studies. Another meta-analysis investigated the correlation between ACT and UFH dose and found a weak correlation (pooled estimate of correlation coefficients 0.132, 95% CI 0.03–0.23), highlighting the need for emerging tools and more appropriate monitoring strategies [46].

Despite its widespread use in clinical settings, ACT has well-documented limitations. While ACT provides a real-time assessment of blood coagulation at the moment of testing, it does not evaluate the strength or stability of the formed clot [6]. Moreover, it has reduced sensitivity at lower levels of anticoagulation, which limits its utility in detecting subtle variations in heparin effects [47]. Moreover, ACT values are susceptible to confounding by numerous physiological and pharmacological variables, including hypothermia, thrombocytopenia, haemodilution, and the concurrent use of antiplatelet agents [4,6,48]. Inter-device and inter-reagent variability further compromise the reliability and reproducibility of ACT measurements across institutions [49]. Despite these limitations, ACT remains a commonly used point-of-care test for anticoagulation worldwide [44].

### 4.2. Activated Clotting Time and Haemorrhagic Events

Currently, no coagulation test reliably predicts bleeding or thrombotic risk in patients receiving ECMO support. Existing data are inconsistent, likely due to the absence of large prospective studies and the influence of confounding patient-related factors.

Four out of the seventeen studies reported an association between higher ACT values and bleeding during ECMO support [15,30,33,38]. Omar et al. found that patients with bleeding complications had significantly longer ECMO durations, leading to prolonged exposure to anticoagulation [33]. This increased the likelihood of supratherapeutic anticoagulant levels and subsequently elevated the incidence of haemorrhage [33]. Bailly et al. reported that higher ACT values on the day before bleeding were associated with an increased likelihood of bleeding [15]. The authors concluded that ACT-guided heparin titration may lower bleeding risk more than thrombosis, with no study showing a clear advantage of ACT over other monitoring methods.

The diverse reporting practices and inherent limitations of ACT restrict its utility. The considerable variability in how ACT values are reported may originate from the lack of clear definitions and standardization in the timing of ACT measurements and reporting, particularly in retrospective studies. Furthermore, it is often not clear whether the ACT values were recorded as an average value over the entire ECMO period, as the lowest/highest value before or after an event, or at any other time period. However, based on the current evidence, ACT does not appear to be a reliable surrogate for monitoring anticoagulation on ECMO.

### 4.3. Activated Clotting Time and Thromboembolic Events

Of the 30 studies, only 14 reported on thromboembolic adverse events, and 2 studies focused primarily on thrombosis. One study found lower anti factor Xa, ACT, and aPTT levels in patients with thrombosis, suggesting that individualized therapy may be preferable to fixed cut-offs. No correlation was observed between UFH and ACT [29].

Interestingly, two studies reported on higher ACT in patients experiencing thrombosis [24,35], without a significant correlation between UFH doses and ACT. This may reflect the adjusted anticoagulation targets in high-risk patients, where therapy is often intensified or reduced based on bleeding thrombotic risk.

Thromboembolic events are underreported, yet they remain one of the most feared complications of ECMO. Their incidence varies depending on ECMO configuration and patient-related factors. Our findings align with those of available meta-analyses, highlighting the main limitation of thrombosis identification in ECMO patients. Its detection is complicated by the complexity of clinical presentations and the lack of standardized radiological investigations or post mortem examinations. To overcome these challenges, standardized definitions, uniform monitoring protocols, and reliable, non-invasive diagnostic tools are essential to improve anticoagulation management and comparability across studies.

### 4.4. Adverse Events and Mortality

Severe cardiogenic or respiratory shock remains associated with a high risk of adverse events and mortality. The available literature mostly consists of smaller retrospective studies reporting a wide range of adverse events, often lacking standardization due to variations in the identification and reporting criteria for complications. Several studies have attempted to identify the incidence of complications and their impact on survival, being limited by their observational nature, diversity in reporting, and lack of clear outcome definitions [50,51,52]. Therefore, we provide the pooled complications rate, which may more accurately reflect the true rate of complications.

Haemorrhage was identified as the most common adverse event, consistent with the literature [50,52,53]. Thromboembolic complications were reported in 9 of 30 studies, suggesting a comparable incidence (16%) with previous works [50,52,53]. However, the real rate of thromboembolic complications remains vague, as it originates from observational and retrospective studies [54,55].

Finally, 51% (pooled value) of patients did not survive to discharge from hospital, which is consistent with previous meta-analyses and ELSO reports (46%, based on 221,723 ECMO runs) [56].

### 4.5. Future Development

Further prospective and randomized trials are necessary to gain a deeper understanding of optimal anticoagulation monitoring in ECMO patients. Despite the limited data, the use of viscoelastic testing and an anti-factor Xa assays is increasingly recommended in clinical practice [4,57,58,59,60,61]. Multimodal monitoring, which combines different monitoring methods, shows promise; however, the broader implementation may be hindered by high costs and longer turnaround times compared to the point-of-care options. Due to the inherent limitations of UFH, alternative anticoagulants are being evaluated, driven by more predictable pharmacokinetics [2,62]. Furthermore, anticoagulation-free ECMO has become an area of active research in recent years [63,64,65].

The introduction of the ELSO bleeding definition represents progress, yet standardized definitions for other adverse events are still lacking. As illustrated by thrombotic events, the absence of clear guidelines impedes consistent reporting and limits data comparability. Establishing standardized protocols is crucial to advancing research quality and improving patient outcomes.

### 4.6. Strengths and Limitations

The strengths of our work include 30 publications involving 2379 patients with ACT-guided ECMO anticoagulation monitoring. Moreover, we controlled for patient overlap in the case of reports from same centres, and we report our results respecting the PRISMA guidelines [10].

However, our work has some limitations. Despite a thorough and organized search, publication and retrieval could occur. It may be possible that certain articles were not published in the databases searched or that some works never became published or accessible. The quality of the provided data is limited by the methodology of the analysed studies, which are predominantly retrospective. Additionally, of the 30 studies analysed, 23 originated from USA, while 7 reported data from other countries. The variation in standards of care across different regions may contribute to additional bias. Most included studies were retrospective in nature and did not consistently report ACT values immediately preceding haemorrhagic or thromboembolic events. Furthermore, the majority of authors did not report whether ACT values were average values over the entire ECMO period or as the lowest or highest values. This lack of consistency in reporting may limit the ability to assess the role of ACT in the development of adverse events.

## 5. Conclusions

Despite extensive research and advancements in ECMO, the optimal approach for anticoagulation monitoring remains undefined. The majority of the studies included in this review did not establish a significant relationship between ACT levels and haemostatic complications in ECMO patients. However, due to its low cost, accessibility, and rapid turnaround time, ACT remains the commonly used method for UFH monitoring in certain centres. Based on the current evidence, ACT does not appear to be a reliable tool for monitoring anticoagulation in patients receiving ECMO, and alternative methods should be considered.

## Figures and Tables

**Table 1:** Characteristics of the included studies (n = 30).

Author Country (Study Period)	Population Study Type	Number of Patients	ECMO Type	ECMO Duration (Days)	Main ECMO Indications	Main Study Aim	**NOS**
Al-Jazairi et al. [12], Saudi Arabia (-)	Adult PR and RT	20	VA: 16 VV: 4	15 (7–28)	Cardiac arrest, intraoperative wean-off failure, acute respiratory failure, bridge to transplant, and others	Correlation between anticoagulation monitoring and UFH infusion dose	Good
Anton-Martin et al. [13], USA (2009–2014)	Paediatric RT	36	VA: 31 VV: 5	154 ^A^ (25–1047)	Pulmonary, cardiac, and eCPR	Predictors for ICH or stroke	Good
Atallah et al. [14], USA (2011–2012)	Adult RT	46	-	11 ± 14.6	Cardiac and respiratory	Evaluation of the correlation between the UFH dose and ACT or aPTT	Good
Bailly et al. [15], USA (2012–2014)	Paediatric PR	481	VA: 400 VV: 81	5.4 (3.0–9.8)	Respiratory, cardiac, and eCPR	Association of anticoagulation practices with bleeding and thrombosis	Good
Deshpande et al. [16], USA (2010–2016)	Paediatric RT	133	VA: 92 VV: 41	-	ARDS, CDH, lower respiratory tract infection, sepsis, etc.	Association of anticoagulation monitoring tools with haemostatic adverse events	Good
Doymaz et al. [17], USA (1997–2010)	Paediatric RT	32	VA: 19 VV: 13	-	Persistent pulmonary hypertension	Incidence and risk factors for ICH	Good
Feih et al. [18], USA (2012–2018)	Adult RT	45	-	99.0 ^A^ (51.0–169.3)	Respiratory failure, cardiogenic shock, and bypass weaning failure	Identification of risk factors for haemostatic complications	Good
Figueroa Villalba et al. [19], USA (2015–2018)	Paediatric RT	65	VA: 50 VV: 15	-	Congenital heart disease, postcardiac surgery, cardiac arrest, respiratory failure, CDH, and others	Effect of monitoring change from ACT to anti-factor Xa	Good
Fitousis et al. [20], USA (2011–2014)	Adult RT	61	VA: 24 VV: 37	244 ± 326.1 ^A^	-	Comparison of the efficacy and safety of aPTT- and ACT-based UFH anticoagulation	Good
Galura et al. [21], USA (2014–2020)	Paediatric RT	27	VA: 24 VV: 3	136 ^A^ (95, 192)	Cardiac and respiratory failure	Comparison of anticoagulation monitoring with ACT to a multimodal strategy (ACT, aPTT, anti-factor Xa, and TEG)	Good
Henderson et al. [22], USA (2013–2015)	Paediatric RT	30	VA: 26 VV: 4	146.8 ± 38.5 ^A^	Cardiac arrest, cardiogenic shock, and respiratory failure	Analysis of anticoagulation goals for predicting haemostatic adverse events	Good
Hong et al. [23], Korea (2017–2019)	Adult RT	43	VA: 31 VV: 12	-	-	Analysis of lower and conventional ACT target (<150 vs. 180–200 s) and its impact on safety and outcome	Good
Irby et al. [24] USA (2009–2011)	Paediatric RT	62	-	-	CBP weaning failure, eCPR, sepsis, respiratory failure, cardiac, bridge to transplant, etc.	Association of anti-factor Xa with ECMO circuit changes	Good
Kasirajan et al. [25], USA (1992–1996)	Adult RT	74	VA: 74	-	Respiratory failure, myocardial infarction, post-cardiotomy, myocarditis, post-heart transplant	Prevalence and risk factors for ICH	Good
Liu et al. [26], China (2019–2020)	Adult RT	17	VA: 11 VV: 6	10 (8, 15)	Respiratory and circulatory support	Comparison between ACT/aPTT and UFH infusion dose	Good
Maul et al. [27], USA (2007–2010)	Paediatric RT	47	-	-	Respiratory or cardiac distress	Comparison of ACT and aPTT for UFH infusion monitoring	Good
Mazzeffi et al. [28], USA (2010–2015)	Adult RT	50	VA: 50	5 (2–8)	Cardiogenic shock, post-cardiotomy shock, and respiratory failure with cardiac dysfunction	Incidence of bleeding and thrombosis (ACT vs. aPTT)	Good
Moynihan et al. [29], Australia (2015–2016)	Paediatric RT	31	VA: 29 VV: 5	144.2 ^A^ (87.3–221.2)	Respiratory failure, sepsis, postoperative cardiac, eCPR, etc.	Correlation between anticoagulation monitoring methods and UFH dose	Good
Nguyen et al. [30], Vietnam (2019–2020)	Adult RT	105	VA: 61 VV: 38 VAV: 6	-	Acute myocarditis, severe anaphylaxis, myocardial infarction, and ARDS	Risk factors for bleeding	Good
Niebler et al. [31], USA (2006–2016)	Paediatric RT	129	-	94.5 ^A^ (59.5–154.5)	Cardiac and noncardiac surgery	Association of ACT and anti-factor Xa with haemostatic complications	Good
Northam et al. [32], USA (2014–2019)	Adult RT	26	VV: 26	5.0 (3.0–9.5)	Acute respiratory distress syndrome	Comparison of multimodal approach (aPTT/anti-factor Xa) and ACT for UFH monitoring	Good
Omar et al. [33], USA (2007–2013)	Adult RT	154	VA: 125 VV: 29	5.7 ± 6.8	eCPR, respiratory failure, pulmonary embolism, cardiogenic shock, post-cardiac or lung surgery, etc.	Predictors and incidence of ICH	Good
O’Meara et al. [34], USA (2012–2012)	Paediatric RT	10	-	-	eCPR, cardiorespiratory failure, and pulmonary hypertension	Change from ACT to anti-factor Xa monitoring and impact on the oxygenator/circuit change	Fair
Perez Ortiz et al. [35], Germany (2018–2019)	Paediatric PR	23	VA; 23	10.3 (1–20)	CDH	Correlation between anticoagulation monitoring methods and UFH dose	Fair
Rama et al. [36], USA (2010–2016)	Paediatric RT	96	VA: 80 VV:16	112 ^A^ (73.4–165.6)	Cardiac, respiratory, and eCPR	Incidence of haemostatic complications based on ACT or anti-factor Xa monitoring	Fair
Reed et al. [37], USA (2004–2008)	Paediatric RT	29	-	-	Congenital or acquired cardiac or pulmonary diseases	Incidence and predictors of haemostatic complications	Fair
Riley et al. [38], USA (2007–2010)	Adult RT	53	VA: 53	-	Post-cardiotomy	Acceptable blood loss and the sensitivity to detect haemorrhage for different coagulation monitoring methods measured in the first hours of ECMO	Good
Saini et al. [39], USA (2011–2012)	Paediatric RT	24	VA: 19 VV: 5	-	Myocarditis, postoperative support, ARDS, pulmonary hypertension, CDH, etc.	Laboratory predictors for haemorrhage and mortality	Good
Shah et al. [40], USA (2009–2014)	Adult RT	53	VV: 53	10 (5–17)	ARDS and bridge to lung transplant	Change in monitoring from ACT to aPTT in relation to haemostatic complications and overall patient outcome	Fair
Yang et al. [41], China (2017–2020)	Paediatric RT	148	VA: 148	-	Congenital heart disease	Gastrointestinal bleeding risk factors	Good

^A^ Data presented in hours. Abbreviations: NOS: Newcastle–Ottawa Scale; ACT: active clotting time; aPTT: activated partial thromboplastin time; ECMO: extracorporeal membrane oxygenation; RT: retrospective; eCPR: extracorporeal cardiopulmonary resuscitation; PR: prospective; VA: venoarterial; VV: venovenous; CDH: congenital diaphragmatic hernia; ARDS: acute respiratory distress syndrome; ICH: intracerebral/cranial haemorrhage; TEG: thromboelastography; RSV: respiratory syncytial virus.

**Table 2:** Mortality and adverse events (n = 30).

Outcome	Number of Studies Reporting Data (Events)	Pooled Rate (95% CI)	I^2^ (*p*-Value)
**Mortality**			
In-hospital mortality	17 (693)	51.3 (44.0; 58.7)	84% (<0.001)
ICU mortality	5 (90)	42.2 (26.6; 59.4)	82% (<0.001)
Death during ECMO	5 (81)	36.8 (21.1; 55.9)	84% (<0.001)
**Bleeding**			
Major bleeding	13 (501)	49.2 (36.7; 61.9)	90% (<0.001)
Any bleeding	17 (700)	47.8 (38.5; 57.2)	88% (<0.001)
Cerebral haemorrhage	15 (218)	13.8 (10.3; 18.3)	73% (<0.001)
Gastrointestinal bleeding	5 (51)	12.1 (5.8; 23.5)	77% (0.001)
Pulmonary bleeding	4 (61)	5.3 (2.1; 12.8)	72% (0.014)
Other bleeding	6 (92)	33.3 (18.3; 52.6)	87% (<0.001)
**Thrombosis**			
Any thrombosis	16 (309)	25.1 (17.6; 34.4)	88% (<0.001)
ECMO circuit and membrane clot	9 (168)	16.6 (10.4; 25.4)	81% (<0.001)
Deep venous thrombosis	3 (12)	12.1 (7.0; 20.2)	16% (0.304)
Limb ischemia	2 (26)	7.5 (1.5; 30.8)	94% (<0.001)
Ischemic stroke	7 (50)	5.5 (4.2; 7.2)	0% (0.465)
Other thrombosis	4 (30)	14.4 (1.5; 65.5)	95% (<0.001)

Abbreviations: ICU, intensive care unit; ECMO, extracorporeal membrane oxygenation.

## Data Availability

The data that support the findings of this study are available upon reasonable request from the corresponding author.

## Supplementary Materials

The following supporting information can be downloaded at https://www.mdpi.com/article/10.3390/jcdd12070267/s1, Table S1: PRISMA 2020 checklist—Preferred Reporting Items for Systematic review and Meta-Analysis; Table S2: Study inclusion and exclusion (PICOS criteria); Table S3: Search strategy; Table S4: Detailed information on the data extraction; Table S5: Anticoagulation monitoring and outcomes of included articles (n = 30).

## Abbreviations

The following abbreviations are used in this manuscript:

ACT	Activated clotting time
aPTT	Activated partial thromboplastin time
ARDS	Acute respiratory distress syndrome
CDH	Congenital diaphragmatic hernia
ECMO	Extracorporeal membrane oxygenation
eCPR	Extracorporeal cardiopulmonary resuscitation
ELSO	Extracorporeal Life Support Organization
ICH	Intracerebral/cranial haemorrhage
ICU	Intensive care unit
NOS	Newcastle–Ottawa Scale
PR	Prospective
PT	Prothrombin time
ROTEM	Rotational thromboelastometry
RSV	Respiratory syncytial virus
RT	Retrospective
TEG	Thromboelastography
UFH	Unfractionated heparin
VA	Venoarterial
VV	Venovenous
