# Supplementary material for: Activated Clotting Time and Haemostatic Complications in Patients Receiving ECMO Support: A Systematic Review
Source: J Cardiovasc Dev Dis. 2025 Jul 13;12(7):267. doi: 10.3390/jcdd12070267 (PMC12296153; doi:10.3390/jcdd12070267)
Supplement: Supplementary file 1 [file jcdd-12-00267-s001.zip › jcdd-3697991-supplementary.pdf]

Journal of Cardiovascular Development and Disease

*Supplementary Materials*

# Activated Clotting Time and Haemostatic Complications in Patients Receiving ECMO Support: A Systematic Review

Contents

**Table S1.** PRISMA 2020 checklist - Preferred Reporting Items for Systematic review and Meta-Analysis [10] ............ 2
**Table S2.** Study inclusion and exclusion (PICOS criteria) ........ 5
**Table S3.** Search strategy ........ 6
**Table S4.** Detailed information on the data extraction ........ 6
**Table S5.** Anticoagulation monitoring and outcomes of included articles (n =30) ........ 7

**Table S1.** PRISMA 2020 checklist - Preferred Reporting Items for Systematic review and Meta-Analysis [10]

| Section and Topic | Item | Checklist item | Location where item is reported |
|---|---|---|---|
| TITLE | | | |
| Title | 1 | Identify the report as a systematic review. | Page 1 |
| ABSTRACT | | | |
| Abstract | 2 | See the PRISMA 2020 for Abstracts checklist. | Page 1 |
| INTRODUCTION | | | |
| Rationale | 3 | Describe the rationale for the review in the context of existing knowledge. | Page 2 |
| Objectives | 4 | Provide an explicit statement of the objective(s) or question(s) the review addresses. | Page 2 |
| METHODS | | | |
| Eligibility criteria | 5 | Specify the inclusion and exclusion criteria for the review and how studies were grouped for the syntheses. | Page 2 |
| Information sources | 6 | Specify all databases, registers, websites, organisations, reference lists and other sources searched or consulted to identify studies. Specify the date when each source was last searched or consulted. | Page 2 |
| Search strategy | 7 | Present the full search strategies for all databases, registers and websites, including any filters and limits used. | Page 2, Supple-mentary |
| Selection process | 8 | Specify the methods used to decide whether a study met the inclusion criteria of the review, including how many reviewers screened each record and each report retrieved, whether they worked independently, and if applicable, details of automation tools used in the process. | Page 3 |
| Data collection process | 9 | Specify the methods used to collect data from reports, including how many reviewers collected data from each report, whether they worked independently, any processes for obtaining or confirming data from study investigators, and if applicable, details of automation tools used in the process. | Page 3 |
| Data items | 10a | List and define all outcomes for which data were sought. Specify whether all results that were compatible with each outcome domain in each study were sought (e.g. for all measures, time points, analyses), and if not, the methods used to decide which results to collect. | Page 2-3, Supple-mentary |
| | 10b | List and define all other variables for which data were sought (e.g. participant and intervention characteristics, funding sources). Describe any assumptions made about any missing or unclear information. | Page 2-3, Supple-mentary |
| Study risk of bias assessment | 11 | Specify the methods used to assess risk of bias in the included studies, including details of the tool(s) used, how many reviewers assessed each study and whether they worked independently, and if applicable, details of automation tools used in the process. | Page 3 |
| Effect measures | 12 | Specify for each outcome the effect measure(s) (e.g. risk ratio, mean difference) used in the synthesis or presentation of results. | Page 3 |

| Section and Topic | Item | Checklist item | Location where item is reported |
|---|---|---|---|
| Synthesis methods | 13a | Describe the processes used to decide which studies were eligible for each synthesis (e.g. tabulating the study intervention characteristics and comparing against the planned groups for each synthesis (item #5)). | Page 3 |
| | 13b | Describe any methods required to prepare the data for presentation or synthesis, such as handling of missing summary statistics, or data conversions. | Page 3 |
| | 13c | Describe any methods used to tabulate or visually display results of individual studies and syntheses. | Page 3 Supplementary |
| | 13d | Describe any methods used to synthesize results and provide a rationale for the choice(s). If meta-analysis was performed, describe the model(s), method(s) to identify the presence and extent of statistical heterogeneity, and software package(s) used. | Page 3-5 |
| | 13e | Describe any methods used to explore possible causes of heterogeneity among study results (e.g. subgroup analysis, meta-regression). | |
| | 13f | Describe any sensitivity analyses conducted to assess robustness of the synthesized results. | |
| Reporting bias assessment | 14 | Describe any methods used to assess risk of bias due to missing results in a synthesis (arising from reporting biases). | Page 3 |
| Certainty assessment | 15 | Describe any methods used to assess certainty (or confidence) in the body of evidence for an outcome. | Page 3 |
| RESULTS | | | |
| Study selection | 16a | Describe the results of the search and selection process, from the number of records identified in the search to the number of studies included in the review, ideally using a flow diagram. | Page 3-9 |
| | 16b | Cite studies that might appear to meet the inclusion criteria, but which were excluded, and explain why they were excluded. | Supplementary |
| Study characteristics | 17 | Cite each included study and present its characteristics. | Page 5-7 |
| Risk of bias in studies | 18 | Present assessments of risk of bias for each included study. | Page 5-7 |
| Results of individual studies | 19 | For all outcomes, present, for each study: (a) summary statistics for each group (where appropriate) and (b) an effect estimate and its precision (e.g. confidence/credible interval), ideally using structured tables or plots. | Page 3-9, Supplementary |
| Results of syntheses | 20a | For each synthesis, briefly summarise the characteristics and risk of bias among contributing studies. | Page 5-7, Supplementary |
| | 20b | Present results of all statistical syntheses conducted. If meta-analysis was done, present for each the summary estimate and its precision (e.g. confidence/credible interval) and measures of statistical heterogeneity. If comparing groups, describe the direction of the effect. | Page 8-9, |

| Section and Topic | Item | Checklist item | Location where item is reported |
|---|---|---|---|
| | 20c | Present results of all investigations of possible causes of heterogeneity among study results. | |
| | 20d | Present results of all sensitivity analyses conducted to assess the robustness of the synthesized results. | |
| Reporting biases | 21 | Present assessments of risk of bias due to missing results (arising from reporting biases) for each synthesis assessed. | |
| Certainty of evidence | 22 | Present assessments of certainty (or confidence) in the body of evidence for each outcome assessed. | |
| DISCUSSION | | | |
| Discussion | 23a | Provide a general interpretation of the results in the context of other evidence. | Page 9-11 |
| | 23b | Discuss any limitations of the evidence included in the review. | Page 9-11 |
| | 23c | Discuss any limitations of the review processes used. | Page 11 |
| | 23d | Discuss implications of the results for practice, policy, and future research. | Page 11 |
| OTHER INFORMATION | | | |
| Registration and protocol | 24a | Provide registration information for the review, including register name and registration number, or state that the review was not registered. | Page 2 |
| | 24b | Indicate where the review protocol can be accessed, or state that a protocol was not prepared. | Page 2 |
| | 24c | Describe and explain any amendments to information provided at registration or in the protocol. | Page 2 |
| Support | 25 | Describe sources of financial or non-financial support for the review, and the role of the funders or sponsors in the review. | Page 12 |
| Competing interests | 26 | Declare any competing interests of review authors. | Page 12 |
| Availability of data, code and other materials | 27 | Report which of the following are publicly available and where they can be found: template data collection forms; data extracted from included studies; data used for all analyses; analytic code; any other materials used in the review. | Page 12 |

**Table S2.** Study inclusion and exclusion (PICOS criteria)

| Parameter | Inclusion criteria | Exclusion criteria |
|---|---|---|
| **Population** | - Patients receiving ECMO support | - Patients receiving other mechanical life support<br>- Potential patient overlapping (i.e. studies from the same center, register studies overlapping with submitting center) |
| **Intervention** | - ECMO<br>- Anticoagulation with UFH<br>- Anticoagulation monitoring using ACT | - Studies not reporting on ACT anticoagulation monitoring<br>- Other or no anticoagulation |
| **Comparator** | None | None |
| **Outcomes** | Primary:<br>- Anticoagulation monitoring using ACT<br>- The association of haemorrhagic events with ACT<br>- The association of thromboembolic events with ACT<br>Secondary:<br>- Reported adverse events<br>- Rate of adverse events<br>- In-hospital mortality | - |
| **Study design** | - Randomized trials,<br>- Prospective and retrospective cohort studies,<br>- Grey literature | - Meta-analyses,<br>- Systematic reviews,<br>- Conference abstracts,<br>- Case reports,<br>- Short reports,<br>- Letters |

*PICOS* patients/population, intervention, comparator, outcomes, study design, *ECMO* extracorporeal membrane oxygenation, *UFH* unfractionated heparin, ACT activated clotting time

**Table S3.** Search strategy

| **Database: PubMed** |
|---|
| Code: ((((extracorporeal membrane oxygenation[MeSH Terms]) OR (ECMO)) OR (extracorporeal life support)) OR (ECLS)) AND ((((((((anticoagulation monitoring) OR (anticoagulation)) OR ("Blood Coagulation Tests"[Mesh])) OR (Activated clotting time)) OR (ACT)) OR (anti Xa)) OR (anti-factor Xa))) Filters: Humans |
| **Database: Scopus** |
| Code: (TITLE-ABS-KEY ("extracorporeal membrane oxygenation" OR ECMO OR "extracorporeal life support" OR ECLS AND "anticoagulation monitoring" OR anticoagulation OR ACT OR "Activated clotting time" OR "anti Xa" OR "anti-factor Xa")) |

**Table S4.** Detailed information on the data extraction

| **Characteristics** | **Description** |
|---|---|
| **Study characteristics** | Author, study design, publication year, number of patients, recruitment period, institution and country, type of ECMO support, and main aim. |
| **Patient and ECMO characteristics** | Age, cannulation type, and ECMO support duration. |
| **Adverse events** | Reported haemorrhagic events: Major bleeding, cerebral haemorrhage, gastrointestinal bleeding, pulmonary bleeding, any bleeding, other bleeding; thrombotic events: ECMO circuit and membrane clot, deep venous thrombosis, limb ischemia, ischemic stroke, any thrombosis, other thrombosis |
| **Mortality** | Mortality during ECMO, critical care unit and hospital stay. |
| **Anticoagulation regime** | Type of anticoagulation, information on monitoring, goal ACT range, and the authors conclusion on association of anticoagulation monitoring with bleeding and/or thrombosis. |

*ECMO* extracorporeal membrane oxygenation, *ELSO* extracorporeal life support organization, *UFH* unfractionated heparin, ACT activated clotting time

Journal of Cardiovascular Development and Disease

MDPI

**Table S5.** Anticoagulation monitoring and outcomes of included articles (n =30)

| Author | Anticoagulation goal (ACT, aPTT or anti-Xa when reported) | ACT measurements per day | Association of ACT with bleeding | Association of ACT with thrombosis |
|---|---|---|---|---|
| Al-Jazairi et al. | ACT: 180-220<br>Anti-factor Xa: 0.3-0.7 or 0.2-0.4 | 3 | No: Patients with multifactorial protocol had less bleeding events than ACT-guided.<br>No (superiority): Multifaced anticoagulation protocol with anti-factory Xa may provide better prediction of UFH dosing than ACT. | |
| Anton-Martin et al. | ACT: 160-195<br>aPTT: 60-80<br>Anti-factor Xa: 0.3-0.7 | 24 | No: The median values of ACT, PTT, and anti-factor Xa before the cerebrovascular event were not significantly different between the haemorrhage group and control (ACT: 24h p = 0.10; 72h p = 0.24). | No: The median values of ACT, PTT, and anti-factor Xa before the cerebrovascular event were not significantly different between the infarct group and control (ACT: 24h p = 0.87; 72h p = 0.46). |
| Atallah et al. | ACT: 140-180 | 24 to 8 | No: ACT greater than 180s was not associated with an increased requirement for packed red blood cells transfusion.<br>No (superiority): There is a little to no correlation between ACT und UFH dose | |
| Bailly et al. | ACT: 160-220<br>Anti-factor Xa: 0.3-0.7 | - | Yes: Higher ACT levels on the day before bleeding were associated with higher odds for bleeding (OR 1.03, 95% CI 1.00, 1.05, p=0.047). | No: Higher ACT did not reduce odds of thrombosis. |
| Deshpande et al. | ACT: 190-220<br>Anti-factor Xa: 0.3-0.7 | - | No: No significant associations between ACT, anti-factor Xa, or aPTT with haemorrhage. | No: No significant associations between ACT, anti-factor Xa, or aPTT with thrombosis. |
| Doymaz et al. | ACT: 200-225<br>aPTT: 80-110 | 24 | No: No difference in ACT in group with and without ICH. | |

| | | | | |
|---|---|---|---|---|
| Feih et al. | ACT: 160-220<br>Anti-factor Xa:<br>0.21-0.35 or<br>0.3-0.7 | - | No (superiority): Anti-Xa–based anticoagulation protocol is associated with a lower hazard of major bleeding compared to an ACT. Antithrombin supplementation was higher in ACT-guided patients | |
| Figueroa Villalba et al. | ACT: 180-220<br>Anti-factor Xa: 0.2-0.4 | Initial 24<br>Followed with 12 | No (superiority): Introducing anti-Xa instead of ACT was associated with decreased clinical episodes of bleeding, circuit changes, blood draws, and costs<br>ACT was not predictive of UFH level. | No: ACT was not predictive of UFH level or need for circuit change. |
| Fitousis et al. | ACT: 140-180 | Initial 24<br>Followed with 8 | No (superiority): ACT is not superior compared to aPTT. ACT-guided group required more blood product transfusions. | |
| Galura et al. | ACT: 180-200<br>aPTT: 64-95<br>Anti-factor Xa: 0.35-0.7 | - | No: Multimodal anticoagulation monitoring (ACT, aPTT, anti-Xa, or TEG) appears superior to ACT-only strategies and may reduce heparin exposure and risk of bleeding<br>No (superiority): | |
| Henderson et al. | Anti-factor Xa: 0.2-.0.7 | 24 | No: ACT did not perform well at predicting a significant bleeding (AUC =0.52; p>0.05). | No: ACT did not perform well at predicting thrombosis (OR 1.02 p =0.496). |
| Hong et al. | ACT: 130-200 | Initial 24<br>Followed with 12 to 6 | | No: Lower ACT was not associated with increased thromboembolic risk; however, oxygenator change was significantly higher in low ACT group. |
| Irby et al. | ACT: 180-220 | 24 | | No: No difference in ACT values between groups with or without circuit/membrane oxygenator change. |
| Kasirajan et al. | ACT: 180-200 | - | No: No significant association between ACT and bleeding (p = 0.55). | |
| Liu et. al | ACT: (VV 160-180; VA 180-200)<br>aPTT: 2-3 times control | | No (superiority): aPTT appears to be a more reliable indicator than ACT.<br>Correlation between ACT and aPTT or UFH is poor, patients with aPTT monitoring had fewer cumulative heparin doses per day and fewer daily heparin dose changes | No: There was no difference between the ACT and aPTT monitoring in terms of embolism occurrence. |

| | | | | |
|---|---|---|---|---|
| Maul et al. | ACT: 180-200<br>aPTT: 70-90 | 24 | No (superiority): aPTT guided anticoagulation led to significant decrease in bleeding compared to ACT ($p < 0.01$). aPTT may be a superior tool over the ACT.<br>ACT showed a weak correlation to UFH ($r = 0.22$) | |
| Mazzeffi et al. | ACT: 180-200<br>aPTT: 60-80 | Initial 12<br>Followed with 8 | No (superiority): There was no significant difference in serious bleeding or thrombosis between the groups. Patients treated with an ACT protocol received more total blood transfusion | No: There was no significant difference in thrombosis between the groups (ACT vs. aPTT). |
| Moynihan et al. | - | 4 | No: No significant association between ACT and bleeding ($p = 0.177$)<br>No (superiority): No correlation between UFH and ACT ($r = -0.08$; $p = 0.056$) | Yes: ACT was lower in patients experiencing thrombosis ($p = 0.003$) |
| Nguyen et al. | ACT: 180-220<br>aPTT: 45-80<br>Anti-factor Xa: 0.3-0.7 | 4 | Yes: ACT > 220s was an independent predictor of major bleeding (aOR 3.90, $p = 0.017$). | |
| Niebler et al. | ACT:<br>140-160, 160-180, or 180-200<br>Anti-factor Xa: 0.5-0.7 | 24 | No (superiority): Less bleeding complications with anti-factor Xa anticoagulation monitoring compared to ACT. | No (superiority): Less thrombotic complications with anti-factor Xa anticoagulation monitoring compared to ACT. |
| Northam et al. | ACT: 180-200<br>aPTT: 1.5-2.5 times control<br>Anti-factor Xa: 0.3-0.7 | 24 | Neutral (superiority): No differences in major bleeding with a multimodal UFH monitoring protocol (aPTT and anti-factor Xa) compared to ACT. Multimodal approach led to reduced antithrombin administration rates and lower heparin infusion rate. | Neutral (superiority): No differences in thrombosis rates with a multimodal UFH monitoring protocol (aPTT and anti-factor Xa) compared to ACT. |
| Omar et al. | | - | Yes: ACT is significantly higher in patients with ICH ($p = 0.016$). | |
| O'Meara et al. | ACT: 160-180<br>Anti-factor Xa:0.4-0.8 | Initial 24<br>Followed with 12 | No (superiority): Increased number of blood draws per day in ACT group, increased number of patients not within the goal range | |
| Perez Ortiz et al. | ACT: 160-180 | 24 | No: No significant association between ACT and bleeding.<br>No (superiority): Anti-factor Xa correlates stronger to UFH dose than ACT. Managing anticoagulation with anti-factor Xa could | No: No significant association between ACT and thrombosis ($p = 0.85$). |

| | | | | |
|---|---|---|---|---|
| | | | reduce thrombotic and haemorrhagic complications compared to ACT | |
| Rama et al. | ACT: 200-240<br>Anti-factor Xa:0.3-0.7 | 24 | Neutral (superiority): The use of anti-factor Xa levels as a basis for anticoagulation management is likely equally as effective and safe as ACT | |
| Reed et al. | ACT: 180-220 | 24 | No: No association between haemorrhage at autopsy and coagulation tests (PT, PTT, platelet count, fibrinogen, ACT, and heparin dose). The amount of time a patient spent at a supratherapeutic ACT, the number of heparin dose changes and the variability of ACTs failed to predict haemorrhage. | No: No association between thrombosis at autopsy and coagulation tests (PT, PTT, platelet count, fibrinogen, ACT, and heparin dose). The amount of time a patient spent at a supratherapeutic ACT, the number of heparin dose changes and the variability of ACTs failed to predict thrombosis. |
| Riley et al. | - | 6 | Yes: ACT was higher in patients with bleeding at 8h (p = 0.017).<br>No (superiority): ACT, aPTT, TEG maximum amplitude significantly predicted non-bleeding with varying degrees of sensitivity (aPTT and TEG maximum amplitude are superior to ACT). | |
| Saini et al. | - | - | No: no significant association between ACT and bleeding (p = 0.43).<br>No (superiority): ACT had the lowest prediction of severe bleeding (AUC 0.56; 0.39–0.75, p = 0.42). | |
| Shah et al. | ACT: 160-180<br>aPTT: 60-80 or 45-55 | - | No (superiority): L-PTT strategy was associated with a reduction in major bleeding events compared with the ACT and red blood cells transfusion usage reduced in L-PTT compared to ACT.<br>Significant reduction in major bleeding in L-aPTT group compared to ACT (p = 0.02). | |
| Yang et al. | ACT: 180-220<br>aPTT: 50-80 | - | No (bleeding): Higher ACT was not an independent risk factor for gastrointestinal bleeding. | |

Abbreviations: ACT: active clotting time; aPTT activated partial thromboplastin time; ICH intracerebral/cranial haemorrhage; TEG thromboelastography;
